# Contribution of the Cerebellum to Predictive Motor Control and Its Evaluation in Ataxic Patients

**DOI:** 10.3389/fnhum.2019.00216

**Published:** 2019-06-26

**Authors:** Shinji Kakei, Jongho Lee, Hiroshi Mitoma, Hirokazu Tanaka, Mario Manto, Christiane S. Hampe

**Affiliations:** ^1^Tokyo Metropolitan Institute of Medical Science, Tokyo, Japan; ^2^Komatsu University, Komatsu, Japan; ^3^Medical Education Promotion Center, Tokyo Medical University, Tokyo, Japan; ^4^Japan Advanced Institute of Science and Technology, Nomi, Japan; ^5^Centre Hospitalier Universitaire de Charleroi, Charleroi, Belgium; ^6^Department of Neurosciences, University of Mons, Mons, Belgium; ^7^School of Medicine, University of Washington, Seattle, WA, United States

**Keywords:** cerebrocerebellar loop, electromyography (EMG), movement kinematics, cerebellar ataxia, viscosity, elasticity

## Abstract

Goal-directed movements are predictive and multimodal in nature, especially for moving targets. For instance, during a reaching movement for a moving target, humans need to predict both motion of the target and movement of the limb. Recent computational studies show that the cerebellum predicts current and future states of the body and its environment using internal forward models. Sensory feedback signals from the periphery have delays in reaching the central nervous system, ranging between tens to hundreds of milliseconds. It is well known in engineering that feedback control based on time-delayed inputs can result in oscillatory and often unstable movements. In contrast, the brain predicts a current state from a previous state using forward models. This predictive mechanism most likely underpins stable and dexterous control of reaching movements. Although the *cerebro-cerebellum* has long been suggested as loci of various forward models, few methods are available to evaluate accuracy of the forward models in patients with cerebellar ataxia. Recently, we developed a non-invasive method to analyze receipt of motor commands in terms of movement kinematics for the wrist joint (*B_r_/K_r_* ratio). In the present study, we have identified two components (F1 and F2) of the smooth pursuit movement. We found that the two components were in different control modes with different *B_r_/K_r_* ratios. The major F1 component in a lower frequency range encodes both velocity and position of the moving target (*higher B_r_/K_r_* ratio) to synchronize movement of the wrist joint with motion of the target in a *predictive* manner. The minor F2 component in a higher frequency range is biased to position control in order to generate intermittent small step-wise movements. In cerebellar patients, the F1 component shows a selective decrease in the *B_r_/K_r_* ratio, which is correlated with decrease in accuracy of the pursuit movement. We conclude that the *B_r_/K_r_* ratio of the F1 component provides a unique parameter to evaluate accuracy of the predictive control. We also discuss the pathophysiological and clinical implications for clinical ataxiology.

## Introduction

Goal-directed movements are predictive in nature, especially for moving targets in the environment of daily life. The prediction is in essence multimodal. For instance, during a reaching task for a moving target, humans need to predict both motion of the target and movement of the limb to match them optimally. Making predictions and validating the predictions against actual sensory information is a fundamental function of the nervous system. Prediction errors and assessment of the discrepancy between predicted and actual information are critical parameters (Popa and Ebner, 2019).

Recent computational studies posit a mechanism that predicts current and future states of the body and its environments by integrating an estimate of previous state and efference copies of motor signals, the computation known as an internal forward model (Wolpert et al., 1995; Miall and Wolpert, 1996; Davidson and Wolpert, 2005). Sensory feedback signals through sensory organs have inevitable delays to reach the central nervous system, between tens to hundreds of milliseconds. It is well known in engineering that feedback control based on time-delayed inputs can result in oscillatory and often unstable movements (Miall et al., 1993b; Kawato, 1999). It is most likely that the brain predicts a current state from a previous state with forward models (Wolpert et al., 1995; Miall and Wolpert, 1996). The cerebellum has been suggested as the locus of the forward-model computation of state prediction from psychophysical (Nowak et al., 2007; Tseng et al., 2007; Synofzik et al., 2008), neuroimaging (Blakemore et al., 2001; Kawato et al., 2003; Schlerf et al., 2012), and non-invasive stimulation (Miall et al., 2007; Lesage et al., 2012) studies in humans and electrophysiological studies (Pasalar et al., 2006; Ebner and Pasalar, 2008) in monkeys (for review, see Shadmehr et al., 2010; Ishikawa et al., 2016). Recently, our group demonstrated that current outputs from the cerebellum (firing rates of dentate cells) contained predictive information about future inputs to the cerebellum (firing rates of mossy fibers), thereby providing a strong support to the forward-model hypothesis of the cerebellum (Tanaka et al., 2019). The computation of a forward model contributes to predictive control in the presence of considerable delays in sensory feedback (Desmurget and Grafton, 2000).

The predictive control (also known as internal feedback) and corrective control (known as sensory feedback) (Lacquaniti et al., 1982; Soechting and Lacquaniti, 1988) together play an integral role in the optimal feedback control (OFC) model (Todorov and Jordan, 2002). The OFC model predicts that the gain in sensory feedback is not prefixed but rather adaptive as reported in psychophysical experiments in response to direction-dependent visual perturbations (Franklin et al., 2014), difference in feedback delays across multiple modalities (Crevecoeur et al., 2016), or imposed external force fields (Franklin et al., 2017; see Crevecoeur and Kurtzer, 2018 for review). The task-dependent modulation of feedback gain is likely processed within transcortical feedback loops between cortical sensorimotor areas, particularly the primary motor cortex, and spinal motor circuits (Pruszynski et al., 2011, 2014; for review, see Scott et al., 2015). In summary, the existing studies indicate a dissociation between the two computational elements in the OFC model: the forward-model computation in the cerebellum, and the sensory-feedback computation in cortical sensorimotor areas (Shadmehr and Krakauer, 2008). We therefore hypothesize that cerebellar patients maintain corrective control based on sensory feedback but suffer from impaired predictive control based on forward-model prediction (Popa and Ebner, 2019).

Although the cerebellum, especially its hemispheric part, has long been suggested as containing loci of various forward models (Wolpert et al., 1998; Bastian, 2006; Miall et al., 2007), there is no reliable method to evaluate accuracy of the forward models in patients with cerebellar ataxia to the best of our knowledge. Our previous studies developed a novel method to analyze relationship between muscle activities and movement kinematics of the wrist joint (Lee et al., 2012, 2013, 2015; Mitoma et al., 2016). We found that the muscle activities for a smooth pursuit movement of the normal control subjects encode both velocity and position of the target, resulting in a precise tracking movement. In contrast, the muscle activities of patients with cerebellar ataxia were characterized by a marked decrease in encoding of velocity and a compensatory increase in encoding of position, resulting in a series of irregular stepwise movements with poor accuracy. In these analyses (Lee et al., 2015), we treated the smooth pursuit movement as a whole (i.e., the entire frequency range) assuming a single controller. In the present study, however, we *reanalyzed* the same data to find that the smooth pursuit movement actually contained *two* distinct components, corresponding to separate frequency bands. We further identified that the two components were in different control modes that corresponded to predictive and corrective control reviewed above, respectively. The *major component* in a lower frequency range (referred to as F1) encodes velocity and position of the moving target in a predictive manner, whereas the *minor component* in a higher frequency range (F2) generates intermittent small step-wise movements to correct positional errors. In cerebellar patients, however, the predictive component is associated with a selective decrease in the velocity component, which results in poorer accuracy of the pursuit movement. The impairment in cerebellar patients was succinctly characterized by a ratio of viscosity to elasticity coefficients (*B_r_/K_r_* ratio defined below) in the F1 component, thereby providing a reliable metric to assess the performance of forward-model prediction. We propose that our new method provides a unique tool to evaluate accuracy of the predictive control in patients with cerebellar ataxia.

## Materials and Methods

### Subjects

Thirteen healthy control subjects with no history of neurological disorders (6 women and 7 men, 44–71 years old, mean = 56.0 years old, all right-handed; see Table 1) and age-matched 19 patients with cerebellar ataxia (12 women and 7 men, 29–77 years old, mean = 60.5 years old, all right-handed; see Table 1) took part in the study. For the patients’ clinical data including Modified Rankin Scale (MRS), see Table 2. All of the subjects were informed of the purpose and procedures of this study in advance and provided written informed consents prior to their participation. The protocol was approved by the ethics committees of the Tokyo Metropolitan Institute of Medical Science and the Tokyo Metropolitan Neurological Hospital. It was conducted in accordance with the ethical standards of the Declaration of Helsinki.

**Table 1:** Characteristics of the control subjects.

Case	#ID	Age
1	Se1	46–50
2	Se2	46–50
3	Se4	61–65
4	Se5	41–45
5	Se6	51–55
6	Se8	51–55
7	Se11	51–55
8	Se12	61–65
9	Se15	56–60
10	Se16	41–45
11	Se19	66–70
12	Se22	66–70
13	Se23	71–75

**Table 2:** Characteristics of the patients with cerebellar ataxia.

Case	#ID	Age	Disease	MRS
1	Ce2	61–65	MSA-C	2
2	Ce3	76–80	CCA	2
3	Ce4	61–65	SCA6	2
4	Ce6	71–75	MSA-C	2
5	Ce8	66–70	CCA	2
6	Ce12	61–65	MSA-C	2
7	Ce16	56–60	SCD	2
8	Ce22	31–35	SCA3	2
9	Ce25	66–70	SCA6	2
10	Ce35	66–70	SCA31	2
11	Ce37	36–40	SCA3	2
12	Ce10	61–65	MSA-C	3
13	Ce11	26–30	SCA3	3
14	Ce15	76–80	CCA	3
15	Ce19	56–60	CCA	3
16	Ce20	56–60	MSA-C	3
17	Ce28	56–60	MSA-C	4
18	Ce1	71–75	MSA-C	4
19	Ce7	56–60	MSA-C	4

*MSA-C, multiple system atrophy (MSA) with cerebellar features; SCD, spinocerebellar degeneration; MRS, Modified Rankin Scale. MRS score – 0: No symptoms at all; 1: No significant disability despite symptoms; able to carry out all usual duties and activities, 2: Slight disability; unable to carry out all previous activities, but able to look after own affairs without assistance, 3: Moderate disability; requiring some help, but able to walk without assistance, 4: Moderately severe disability; unable to walk without assistance and unable to attend to own bodily needs without assistance, 5: Severe disability; bedridden, incontinent and requiring constant nursing care and attention.*

### Experimental Setup and Movement Task

The apparatus and experimental setup were the same as those described in our previous study (see Lee et al., 2008, 2015 in detail). Briefly, the subject sat on a chair approximately 60 cm in front of a monitor that displayed a cursor and a target, and grasped a Strick–Hoffman type manipulandum (Hoffman and Strick, 1999; Lee et al., 2015, Hoyo Elemec Co., Ltd., Sendai, Japan) with his/her right hand. The forearm was supported with an armrest. The cursor was a black dot that moved in proportion to movement of the subject’s wrist. The central position of the manipulandum corresponded to the center of the monitor, and the cursor moved left for flexion, right for extension, up for radial deviation, and down for ulnar deviation. The target was displayed as an open circle whose inside diameter corresponded to 4.5° of wrist movement.

**FIGURE 1:**
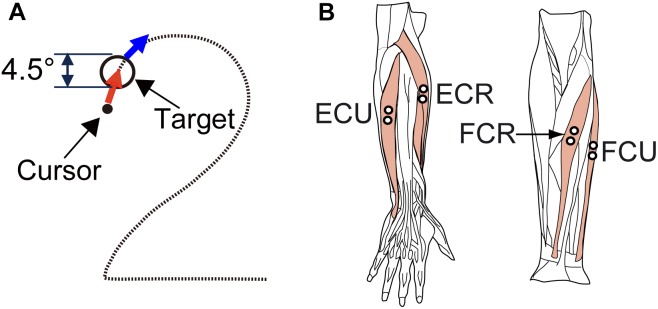
Experimental design. **(A)** Arrangement of the target (circle) and required movement (red arrows) of the cursor (black dot) for the smooth pursuit tasks. The diameter of the targets was equivalent to 4.5 degrees of wrist movement. For the smooth pursuit task, the subjects were required to maintain the cursor within the target. **(B)** The four wrist prime movers from which EMG activity was recorded. ECR, *extensor carpi radialis*; ECU, *extensor carpi ulnaris*; FCU, *flexor carpi ulnaris*; FCR, *flexor carpi radialis*. We recorded the activity of the *extensor carpi radialis longus* (ECRL) and *extensor carpi radialis brevis* (ECRB) together as ECR because these two muscles are indistinguishable with surface electrodes.

**FIGURE 2:**
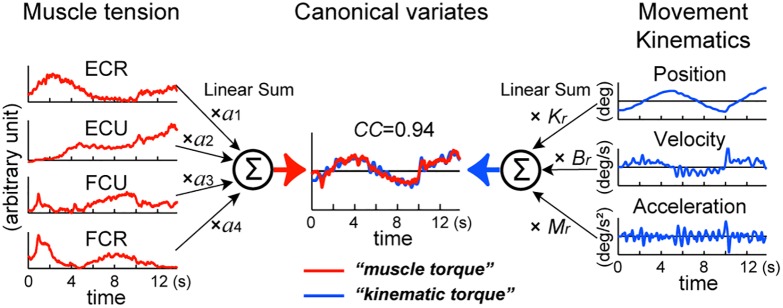
Identification of the relationship between muscle tension and movement kinematics modeled in equation (1). The left panel represents the middle of equation (1). EMG activities of the four muscles (ECR, ECU, FCU, FCR) converted into *muscle tension* (*red lines*) are linearly summed (Σ) after multiplying parameter *a*_1_–*a*_4_, respectively to obtain *muscle torque* in the center (*red line*) (see section “Materials and Methods”). The right panel represents the right side of equation (1). Acceleration (Ax), velocity (Vx), and position (X) of the wrist joint (*blue lines*) are summed (Σ) after multiplying the inertia parameter (*M*), the viscous coefficient (*B_r_*) and the elastic coefficient (*K_r_*), respectively to obtain *kinematic torque* in the center (*blue line*) (see section “Materials and Methods”). We used a canonical correlation analysis (CCA) to obtain values of these parameters. The two canonical variates, *muscle torque* and *kinematic torque*, were calculated by substituting the values for the fitting parameters in equation (1). Note the high canonical correlation (*CC* = 0.94) between the two canonical variates (in *Estimated torque*). This figure explains calculation of torque around the *x*-axis, but the same method applies to calculation of torque around the *y*-axis. Modified from Lee et al. (2015).

The subjects were asked to perform the smooth pursuit task of the wrist joint (Figure 1A) employed in our previous study (Lee et al., 2012, 2015). Each subject was asked to perform a smooth pursuit movement of the wrist joint for a target moving at a constant speed (Figure 1A). To start a trial, the subject placed the cursor within the target, which was stationary at the upper left (*X* = −10°, *Y* = 8°) of the monitor. After a fixed hold period (4 s), the target started moving along the path of the Figure 2 at a constant speed (6.2°/s). The subject was requested to maintain the position of the cursor inside of the moving target as much as possible. After repeating practice three times, each subject performed the task five times. The path of the target was not visible to the subject during the task, however, he/she had some knowledge about the movement of the target thanks to the practice trials.

### Data Acquisition

During the task, we recorded the wrist position (X and Y) and muscle activities [electromyography (EMG) signals] from four wrist prime movers [*flexor carpi radialis* (FCR), *flexor carpi ulnaris* (FCU), *extensor carpi ulnaris* (ECU), and *extensor carpi radialis* (ECR)] (Figure 1B).

We recorded the EMG signals with Ag-AgCl electrode pairs spaced 10 mm apart. EMG signals were amplified (×100,000) and band-pass filtered (150–30,000 Hz) using an amplifier (AB-611J, Nihon Kohden, Tokyo, Japan), and sampled at 2 kHz. The typical locations of the recording electrodes are shown in Figure 1B. The position of each electrode pair was adjusted to maximize the activities of the wrist movements and to minimize those of the finger muscles.

The EMG signals were rectified and filtered with a second-order low-pass filter (cut-off frequency, 3.0 Hz; Mannard and Stein, 1973; Koike and Kawato, 1995) to estimate the muscle tensions from the surface EMG signals (Mannard and Stein, 1973; Koike and Kawato, 1995; Shin et al., 2009; Standenmann et al., 2010). Muscle tension was normalized using a simple normalization technique that sets the amplitude of the muscle tension for 0.78 Nm of isometric wrist joint torque as one (Shin et al., 2009). Finally, we subtracted the normalized muscle tension at the center from the normalized tension to set the tension at the central position to zero. We used the processed muscle tension of the four muscles to estimate wrist joint torque.

### Wrist Joint Model and Identification of the Relationship Between Muscle Activities and Movement Kinematics

We assumed that, if the activity of the wrist muscles determines movement of the wrist joint, it is possible to estimate the wrist joint torque that is calculated from the equation of motion with the activities of the four muscles [equation (1)].

(1) $$\tau (t)=\sum_{i=1}^{4}a_{i}T_{i}(t)=M\ddot{\theta }(t)+B\ddot{\theta }(t)+K\theta (t)$$

where τ(*t*) represents the wrist joint torque. *T_i_* represents muscle tension processed as explained above (see Figure 2: muscle tension: ECR, ECU, FCU, and FCR) and *a_i_* represents the coefficients that convert muscle tension into wrist joint torque (see left side of Figure 2). *a_i_*’s are the moment arm with plus or minus sign according to the pulling direction (i.e., direction of the mechanical action) of each muscle (Lee et al., 2015). The variables θ(*t*), $\dot{\theta }$(*t*), and $\ddot{\theta }$(*t*) represent the angle, angular velocity, and angular acceleration of the wrist joint, respectively. *M*, *B*, and *K* are the inertia parameter (kgm^2^), the viscous coefficient (Nms/rad) and the elastic coefficient (Nm/rad).

Equation (1) is justified if there is a high correlation between the wrist joint torque that is calculated from the movement kinematics [*kinematic torque*: right-hand side of equation (1)] and the wrist joint torque that is calculated from the muscle activities [*muscle torque*: middle of equation (1)]. To identify the relationships between the muscle activities and the movement kinematics for the pursuit task, it is necessary to find the two sets of parameters *a_*1*_*, *a_*2*_*, *a_*3*_*, *a_*4*_*, and *M*, *B*, *K* that optimize the match between the kinematic torque and the muscle torque. We used canonical correlation analysis (CCA) (Härdle and Simar, 2003) for the muscle activities, i.e., [*T_*1*_*(t), *T_*2*_*(t), *T_*3*_*(t), *T_*4*_*(t)], and the movement kinematics, i.e., [$\ddot{\theta }$(*t*), $\dot{\theta }$(*t*), θ(*t*)] in each subject with SAS (University Edition, Release: 3.1, SAS Institute Inc., Cary, NC, United States). The program yielded two parameter vectors (*a_*1*_, a_*2*_, a_*3*_, a_*4*_*), and (*M, B, K*) such that the pair of canonical variates (*a*_1_, *a*_2_, *a*_3_, *a*_4_) [*T*_1_(*t*), *T*_2_(*t*), *T*_3_(*t*), *T*_4_(*t*)]^T^ $[=\sum_{i=1}^{4}a_{i}T_{i}(t)]$ and (*M*, *B*, *K*) [$\ddot{\theta }$(*t*), $\dot{\theta }$(*t*), θ(*t*)]^T^ [= *M*$\ddot{\theta }$(*t*) + *B*θ(*t*) + *K*θ(*t*)] maximize their correlation [i.e., canonical correlation (CC)] (see Figure 2). In the analysis, we used the “NOINT” option that omits subtraction of means from the data, because the muscle activities are always positive or zero. It should be noted that, using CCA, it is not possible to determine absolute values of *M*, *B*, or *K*. Instead, we can obtain their ratios. Therefore, in the following part of this paper, we use *M_r_*, *B_r_*, *K_r_* instead of *M*, *B*, and *K* to emphasize that we focus only on their ratios (see Lee et al., 2015 for discussion).

Furthermore, in our previous study (Lee et al., 2015), we demonstrated a negligible contribution of the acceleration term in equation (1). Therefore, we can simplify the wrist joint model of equation (1) to get equation (2) by removing the acceleration term, at least for the present experimental setup, without sacrificing accuracy of analysis.

(2) $$\sum_{i=1}^{4}a_{i}T_{i}(t)\approx B_{r}\dot{\theta }(t)+K_{r}\theta (t)$$

### Data Analysis

#### Calculation of *B_r_/K_r_* Ratio

In the pursuit task in which the subjects tracked a smooth motion of the target, the joint torque was characterized by the velocity-dependent term and the position-dependent term in Eq. (2). Therefore, we introduce a metric to characterize the contributions of velocity and position as a ratio of the viscous coefficient (*B_r_*) to the elastic (*K_r_*) coefficient: *B_r_/K_r_*. To obtain *B_r_* and *K_r_*, we used the equation (2) and CCA as mentioned above, and calculated *B_r_/K_r_ ratio*. See section “Different *B/K* Ratios for the Two Components of the Pursuit Movements” in the results for more detail.

#### Frequency Analysis of the Wrist Movement

To analyze components of the wrist movement (see section “Two Components of Motor Commands for the Pursuit Movements” and “Different *B/K* Ratios for the Two Components of the Pursuit Movements” in the results for more detail), we performed a frequency analysis (*Fast Fourier Transformation, FFT*) for the velocity of the movement. The wrist movement was decomposed into a low-frequency (≤0.5 Hz) component and a high-frequency (>0.5 Hz) component, referred to as the F1 and F2 components, respectively. The *B_r_/K_r_* ratio, defined above, was computed for the F1 and F2 components separately.

#### Calculation of Delay of the Wrist Movement From the Target Motion

To determine the delay of the wrist movement from the target motion, we searched for the optimal delay that provided the best match between the target motion and the wrist movement. The best match was identified when a delay provided the highest *R*^2^-value for the cross-correlation analysis. See section “Functional Characterization of the F1 Component of the Pursuit Movements” in the results for more detail.

#### Calculation of Errors in Pursuit of the Target

We evaluated the accuracy of the pursuit movement (i.e., motor error) as a sum of instantaneous difference (i.e., distance in degree) between target position and cursor position of the F1 component throughout the trial. We name it an F1 error.

### Statistical Tests

Statistical tests were made using two-sample *t*-test [*t-test2* function in the statistics toolbox of Matlab, Ver. 7.11.0.584 (R2010b), Mathworks, Natick, MA, United States] or Mann–Whitney *U* test [*ranksum* function in the statistics toolbox of Matlab, Ver. 7.11.0.584 (R2010b), Mathworks, Natick, MA, United States].

## Results

### Identification of the Relationship Between Movement Kinematics and Muscle Activity With CCA

**FIGURE 3:**
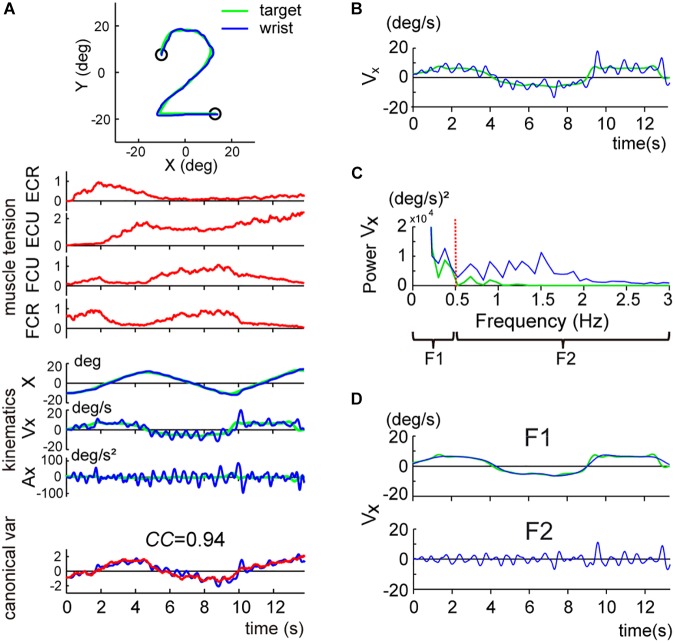
Two components of the smooth pursuit movement. The color convention is the same as in Figure 2. **(A)** Identification of the relationship between muscle activities and movement kinematics in a control subject for the pursuit task. The *blue line* and the *green line* in the *top inset* indicate the *wrist* movement and the *target* motion in a single trial, respectively. *Muscle tension* traces [the top four traces (*red lines*)] show the normalized muscle tension of the four wrist prime movers: ECR, ECU, FCU, and FCR. *Kinematics* traces [the middle three traces (*blue lines*)] show the horizontal (i.e., *x*-axis) components of the movement kinematics: angle (X), angular velocity (Vx), and angular acceleration (Ax), respectively. The *green lines* indicate the kinematics of the target motion. *Canonical var.* traces (the bottom traces) depict the two canonical variates [i.e., *muscle torque* (*red line*)] and *kinematic torque* (*blue line*). The high canonical correlation *CC’s* for the two canonical variates indicate the similarities of the two estimates. The *B_r_/K_r_* ratio for the CCA was 1.35. The same color convention applies to Figure 4, 6. This panel used the same data as Figure 4B in Lee et al. (2015). **(B)** X component of the angular velocity (V_x_) of the smooth pursuit wrist movement (*blue line*) and the target (*green line*). **(C)** Frequency analysis of angular velocity. Note that the target motion (*green line*) has little power above 0.5 Hz (*red vertical dotted line*), while the wrist movement (*blue line*) has components below and above 0.5 Hz. Wrist movement was, therefore, separated into two frequency ranges: F1 (0–0.5 Hz) (*top*) and F2 (0.5–3 Hz) (*bottom*). **(D)** Separation of the angular velocity of the smooth pursuit wrist movement into the two frequency domains, F1 and F2 (*blue lines*). The *green line* indicates the motion of the target. Note that the F1 domain of the wrist movement nearly matches the target motion.

We used CCA to analyze the causality relationship between the muscle activities and the movement kinematics of the wrist joint in 13 control subjects using the wrist joint model (2). With CCA (Figure 2), we obtain the two sets of parameters *a*_1_, *a*_2_, *a*_3_, *a*_4_, and *B_r_, K_r_* that maximize the CC between the two canonical variates (i.e., *muscle torque* and *kinematic torque*) (Figure 2 *middle*). Figure 3A shows a typical example of the relationships during the task, for a control subject. We obtained a precise match between *muscle torque* {i.e., (*B_r_, K_r_*) [$\dot{\theta }$(*t*), θ(*t*)]^T^} (Figure 3A, red lines in *canonical variate*) and *kinematic torque* {i.e., (*B_r_, K_r_*) [$\dot{\theta }$(*t*), θ(*t*)]^T^} (Figure 3A, blue lines in *canonical variate*) with high values of canonical correlation (*CC*s) (*CC* = 0.94). For all control subjects, the average *CC* was 0.93 ± 0.01 (range: 0.91–0.95, *n* = 13) for the pursuit task.

### Two Components of Motor Commands for the Pursuit Movements

When we examined the kinematics of the pursuit movement more closely, we noticed that the velocity profile of the wrist (Figure 3B, blue line) was largely correlated with the smooth velocity profile of the target (Figure 3B, green line), with additional smaller and somewhat vibratory movement of the wrist. This dual pattern was common for all control subjects. To analyze the components of the wrist movement in more detail, we performed a frequency analysis for the velocity of the movement (Figure 3C). The velocity of the pursuit movement was clearly separated into two components: a major component with lower frequency (≤0.5 Hz) and a minor component with higher frequency (>0.5 Hz). The lower frequency component was apparently related to the target motion *per se* (Figure 3D, *F1*) [i.e., most of the power for the target motion (green solid line) was left of the red dotted line (0.5 Hz) in Figure 3C]. Indeed, the wrist movement in the lower frequency range (F1 domain, 0–0.5 Hz; blue line in Figure 3D, *F1*) almost perfectly matched with the target motion (green line in Figure 3D, *F1*). In contrast, the higher frequency component (F2 domain, 0.5–3 Hz, blue line in Figure 3D, *F2*) corresponded to the vibratory wrist movement, which was not correlated with the target motion (green line in Figure 3D).

### Different *B/K* Ratios for the Two Components of the Pursuit Movements

**FIGURE 4:**
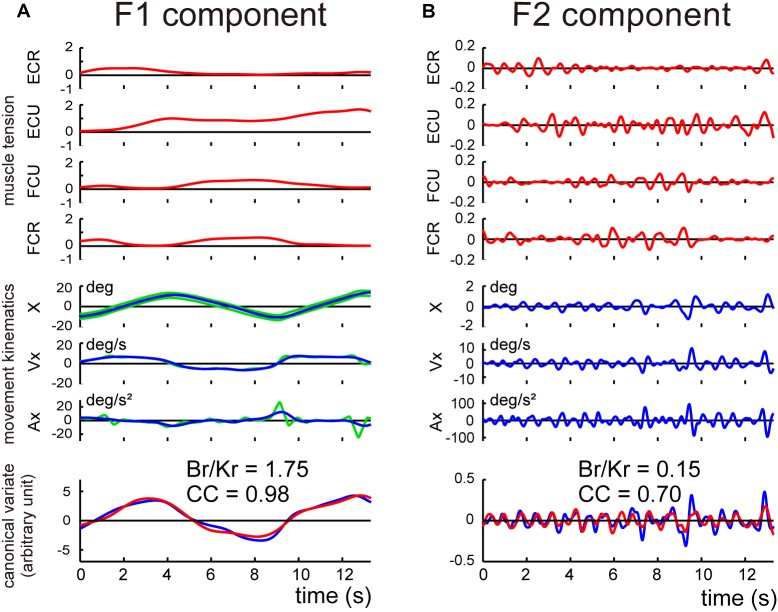
Identification of the relationship between muscle activities and movement kinematics for F1 component **(A)** and F2 component **(B)** of the movement of the control subject shown in Figure 3A. The same convention as in Figure 3A. The high canonical correlation *CC’s* for the two estimated torques in both **(A,B)** indicate the similarities of the two estimates.

**FIGURE 5:**
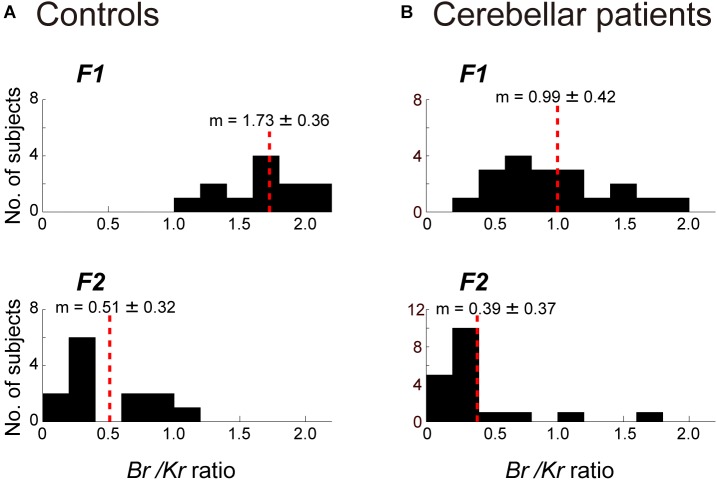
Comparison of the *B_r_/K_r_* ratios for the F1 and F2 components between the controls **(A)** and the cerebellar patients **(B)**. **(A)** *B_r_/K_r_* ratios of the control subjects for the F1 component (top) and the F2 component (bottom) (*n* = 13). Note the highly significant difference for the two components. **(B)** *B_r_/K_r_* ratios of the patients (Table 2, *n* = 19) for the F1 (top) and the F2 (bottom) components (*n* = 19). Note the selective decrease of *B_r_/K_r_* ratios for the F1 component in the patients.

Next, we separated the movement kinematics and activity of each of the four muscles into F1 and F2 components (Figure 4A,B). We then identified the relationship between the muscle activities and movement kinematics for the F1 and F2 components separately with CCA. Figure 4A,B provide examples of the relationship for F1 and F2 of the same trial shown in Figure 3A. Movement kinematics and muscle activity were fairly well correlated for F1 and F2, respectively (*CC* for F1 = 0.98, *CC* for F2 = 0.70). Surprisingly, the *B_r_/K_r_* ratios were different for the two components. In this example, the muscle activity for the lower frequency range (F1) demonstrated a higher *B_r_/K_r_* ratio (1.75), while the muscle activity for the higher frequency range (F2) demonstrated a much lower *B_r_/K_r_* ratio (0.15). The clear dissociation of *B_r_/K_r_* ratios for F1 and F2 components were common for the other trials and for the other control subjects. As illustrated in Figure 5A, muscle activity of the F1 domain (0–0.5 Hz) demonstrated higher *B_r_/K_r_* ratios (1.4–2.5, mean ± SD = 1.84 ± 0.28, *n* = 13) than the pursuit wrist movement as a whole [see Figure 9, in Lee et al. (2015), 0.86–1.91, mean ± SD = 1.30 ± 0.27, *n* = 10]. Thus, the major muscle activity in the F1 domain encoded both velocity and position of the wrist to reproduce the motion of the target. In contrast, muscle activity of the F2 domain (0.5–3 Hz) demonstrated low *B_r_/K_r_* ratios (0.1–1.0, mean ± SD = 0.51 ± 0.32, *n* = 130) (Figure 5A, *F2*), like muscle activity for the step-tracking movement [see Figure 9, in Lee et al. (2015), 0.03–0.28, mean ± SD = 0.17 ± 0.06, *n* = 10]. In other words, the minor muscle activity of the F2 domain appeared to be concerned with frequent small adjustments of wrist position.

### Functional Characterization of the F1 Component of the Pursuit Movements

**FIGURE 6:**
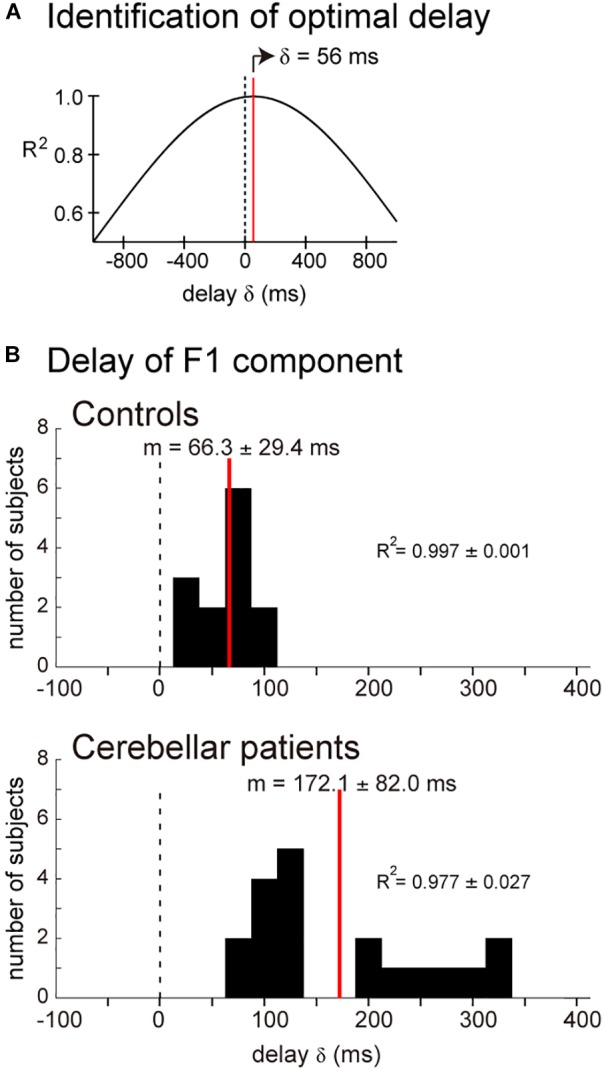
Similarity of the target motion and the F1 domain of the wrist movement. Cross-correlation was calculated by changing delay δ of the target motion relative to the wrist movement. **(A)** Relationship between δ and *R^2^* for the cross-correlation. The optimal δ (59 ms) was determined as the δ for the highest *R_2_*. **(B)** *Controls*: Histogram of the optimal delay δ for the control subjects (*n* = 13). *Cerebellar patients*: Histogram of the optimal delay δ for the cerebellar patients (*n* = 19).

Muscle activity for the pursuit wrist movement consisted of two components with different *B_r_/K_r_* ratios, and the two components appeared to play distinct roles in the pursuit movement. The F1 component appeared to play the primary role to synchronize the movements of the wrist and the motion of the target. To test this hypothesis, we calculated the cross-correlation of the target motion and the F1 component of the wrist movement. As demonstrated by one control subject (Figure 6A), the target position led the wrist movement, but the lead time was very short (56 ms). The average lead time was 47.5 ms for 13 control subjects (mean ± SD = 66.3 ± 29.4 ms, *n* = 13) (Figure 6B, *Controls*). This short lead time means that the F1 component of the wrist movement cannot be generated with a visuomotor feedback control of the target motion, because the conduction time of the peripheral motor nerve (∼10 ms) and electromechanical delay (∼50 ms) alone would take that long. Thus, the delay was too short to be a visuomotor feedback delay. Rather, generation of the motor command in the CNS *must have preceded* the corresponding motion of the target, if we take the average lead time of neuron activity in the motor cortex of the monkey for the wrist movement (∼100 ms) into account (Kakei et al., 1999, 2003).

### Decrease in *B_r_/K_r_* Ratio of the F1 Component in Cerebellar Patients

**FIGURE 7:**
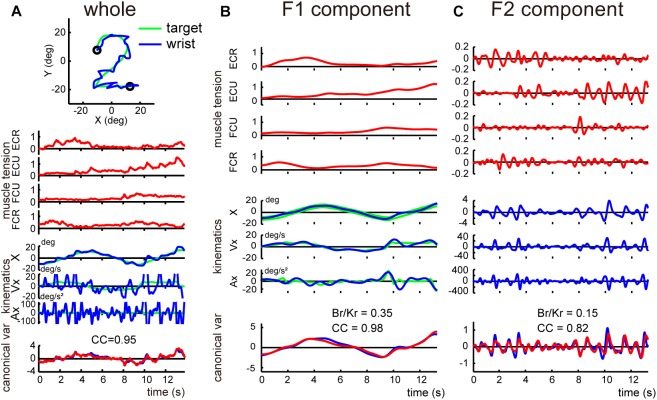
Analysis of the F1 and the F2 components in a cerebellar patient. **(A)** Identification of the relationship between muscle activities and movement kinematics for the *whole* wrist movement. Note a highly ataxic wrist movement shown in the top inset. The same convention as in Figure 3A. This panel used the same data as Figure 5B in Lee et al. (2015). **(B,C)** Identification of the relationship between muscle activities and movement kinematics for the F1 component **(B)** and the F2 component **(C)** of the movement shown in **(A)**. The same convention as in Figure 4. The high canonical correlation *CC’s* for the two estimated torques in both **(B,C)** indicate the similarities of the two estimates.

Next, we determined the *B_r_/K_r_* ratios separately for F1 and F2 components for the cerebellar patients. Figure 7 demonstrates the relationship between movement kinematics and activity of the four muscles for the whole wrist movement (A) and F1 (B) and F2 (C) components in a cerebellar patient. Movement kinematics and muscle activities demonstrated considerably strong canonical correlation for both F1 and F2 components (*CC* for F1 = 0.98, *CC* for F2 = 0.82). Nevertheless, the dissociation of *B_r_/K_r_* ratios for the two components observed in the control subject (Figure 4) was significantly different due to the selective decrease in *B_r_/K_r_* ratios for the F1 component (Figure 7B). The *B_r_/K_r_* ratio for F1 (Figure 7B) was no more than 0.35 and therefore much lower than that observed in the control subject (Figure 4A, *B_r_/K_r_* = 1.75), while the *B_r_/K_r_* ratio for F2 (Figure 7C, *B_r_/K_r_* = 0.15) was the same as that of the control subject (Figure 4B, *B_r_/K_r_* = 0.15).

The marked decrease in *B_r_/K_r_* ratios for the F1 component and relative preservation of low *B_r_/K_r_* ratios for the F2 component were shared by all cerebellar patients (Figure 5B). *B_r_/K_r_* ratios of the F1 component for the cerebellar patients (0.3–1.9, mean ± SD = 0.99 ± 0.42) (Figure 5B, *F1*) were significantly lower than those of the control subjects (1.4–2.5, mean ± SD = 1.84 ± 0.28) (Figure 5A, *F1*) (*p* < 0.001). In contrast, *B_r_/K_r_* ratios of the F2 component were comparable for both groups (compare Figure 5B, *F2* and Figure 5A, *F2*). To summarize, the poor performance of target tracking in the cerebellar patients was attributed to the selective decrease in *Br/Kr* ratios for the F1 component (Figure 5B).

It should be noted that the decrease in *B_r_/K_r_* ratios is not the only anomaly of the F1 component in the cerebellar patients. When we calculated the delay of the F1 component relative to the target motion for the cerebellar patients (Figure 6B, *Cerebellar patients*), we found that the F1 component of the patients was delayed on average by about 100 ms (79.5–322.4 ms, mean ± SD = 172.1 ± 82.0 ms) than that of the controls (15.0–107.4 ms, mean ± SD = 66.3 ± 29.4 ms) (*p* < 0.0001).

### Relationship Between *B_r_/K_r_* Ratio of the F1 Component and Accuracy of Predictive Control

**FIGURE 8:**
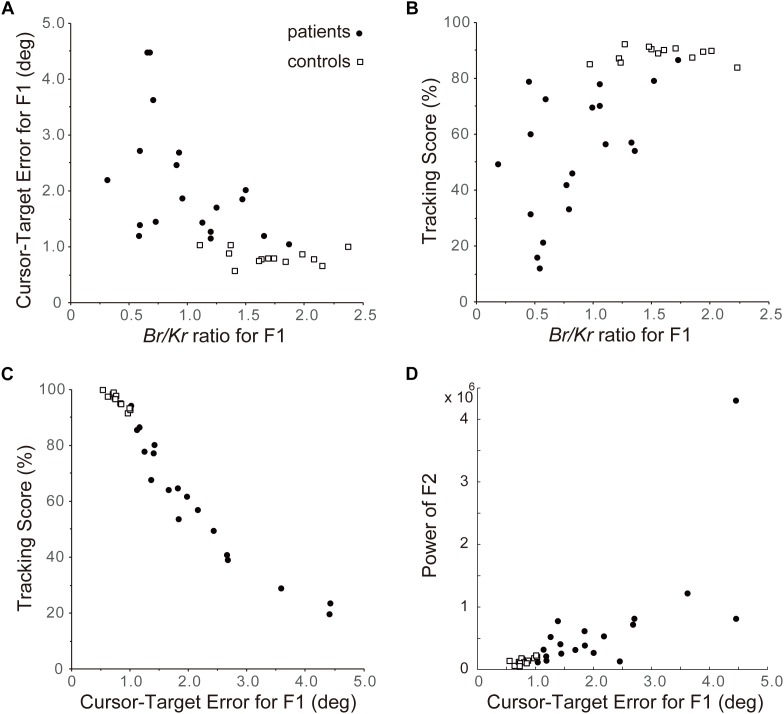
Importance of F1 component of the pursuit movement to determine accuracy of control. **(A)** Relationship between the *B_r_/K_r_* ratios for F1 component and Cursor-Target error for F1 (*F1 error*, in short). The Cursor-Target error for F1 (F1 error) is defined as an average error between the target motion and the F1 component of the movement during a trial. Note the negative correlation. **(B)** Relationship between the *B_r_/K_r_* ratios for F1 component and Tracking Score. Tracking Score is defined as percentage of time when the cursor is kept within the target during a trial. Note the positive correlation. **(C)** Relationship between Cursor-Target Error for F1 (F1 error) and Tracking Score. Note the linear relationship. Overall, *B_r_/K_r_* ratio for F1 component has a strong positive correlation with accuracy of pursuit movement. In other words, poor performance of the cerebellar patients is ascribed to their lower *B_r_/K_r_* ratio for F1 component. **(D)** Relationship between Cursor-Target Error for F1 (F1 error) and power of F2 component. Note the positive correlation.

Next we examined the relationship between *B_r_/K_r_* ratios of the F1 component and performance of pursuit movement in the cerebellar patients and the control subjects (Figure 8). As shown in Figure 4, 6, 7, the F1 component of the pursuit movement is closely related to the predictive component of the movement. Therefore, the characteristic decrease in *B_r_/K_r_* ratio of the F1 component in the cerebellar patients may be an outcome of deterioration of predictive motor control. To test this hypothesis, we examined the relationship between the *B_r_/K_r_* ratio of the F1 component and accuracy of the pursuit movement (i.e., F1 error, see section “Data Analysis”). As shown in Figure 8A, *B_r_/K_r_* ratio of the F1 component and the F1 error demonstrated a clear negative correlation, although the F1 error showed little decrease for higher *B_r_/K_r_* ratio (>1.5). In other words, relative decrease of muscle activity proportional to velocity resulted in poorer accuracy of tracking. However, there remains a possibility that an increase in F1 error (i.e., prediction error) may be compensated by a feedback control and does not affect the overall performance of the pursuit movement. In order to test this possibility, we examined the relationship between the *B_r_/K_r_* ratio of the F1 component and the tracking score. The tracking score is defined as a percentage of time when the cursor was kept within the target in a single trial (Figure 8B). The *B_r_/K_r_* ratio of the F1 component and the tracking score demonstrated a clear positive correlation, although the tracking score showed little increase for higher *B_r_/K_r_* ratios (>1.5). Furthermore, the F1 error and the tracking score demonstrated a strikingly linear (negative) correlation (Figure 8C). In summary, the F1 error is the primary determinant of the overall accuracy of the pursuit movement and a parameter to measure accuracy of the F1 component alone. Overall, *B_r_/K_r_* ratio of the F1 component is a parameter that represent overall accuracy of the pursuit movement.

Finally, we have examined a possibility that the F2 component could be related to an error-correction mechanism. The power (i.e., amount) of F2 component (see Figure 3C, F2) and the F1 error demonstrated a clear positive correlation (Figure 8D, *R*^2^ = 0.53), suggesting that the F2 component is recruited to compensate for increase in F1 error.

## Discussion

We demonstrate that the smooth pursuit movement of the wrist joint consists of two components with distinct *B_r_/K_r_* ratios in control subjects. The major F1 component with higher *B_r_/K_r_* ratio appears to play the primary role to reproduce both velocity and position of the target motion in a *predictive* manner. In contrast, the minor F2 component with lower *B_r_/K_r_* ratio encodes mostly position of small step-wise movements. Therefore, the two control modes, predictive control based on the forward-model prediction and corrective control based on sensory feedback, were identified as the F1 and F2 components, respectively. In cerebellar patients, the predictive F1 component demonstrates a selective decrease in the *B_r_/K_r_* ratio. Notably, the *B_r_/K_r_* ratios of the F1 component has a strong correlation with accuracy of the pursuit movement. In contrast, there was no significant difference between the *B_r_/K_r_* ratios of the F2 component for control and patient groups. Taken together, our results support the hypothesis that cerebellar patients have an impairment in the forward-model prediction while maintaining corrective control in response to sensory feedback. In the following sections, we will focus on five points: (1) dissociation of two components of pursuit movement; (2) functional interpretation of the *B_r_/K_r_* ratio; (3) the *B_r_/K_r_* ratios for F1 and F2 components in patients with cerebellar ataxia and the role of the cerebellum in predictive control; (4) the F1 (*predictive*) component of the pursuit movement and precision of motor control; (5) quantitative evaluation of motor function of patients with cerebellar ataxia based on the *B_r_/K_r_* ratio.

### Dissociation of Two Components of Pursuit Movement

The basic design of this study owes to Beppu et al. (1987) and Miall et al. (1993a). They have examined a specific type of tracking movement in which a visual target moves slowly and smoothly in a predictive manner. They recognized two components of movement during the smooth-tracking movement. The primary component is in lower frequency and the secondary component is in higher frequency and intermittent. They concluded that the lower frequency component reproduces the smooth target motion and the higher frequency/intermittent component represents feedback control. We reasoned that the outputs from the two controllers could be separated with a Fourier transformation due to the differences in the frequency ranges of the two components. In the present study, we established a new method to separate the outputs from the two controllers and to evaluate the accuracy of the predictive controller (Figure 3, 4). We further applied the method to evaluate the predictive controllers in patients with cerebellar ataxia (Figure 7, 8). Our novel finding was that the F1 component was predictive of the target motion and was selectively impaired in the cerebellar patients (Figure 5B, 6B).

### Functional Interpretation of the *B_r_/K_r_* Ratio

In our previous study (Lee et al., 2015), we established a simple linear model for the wrist joint to analyze the causal relationship between muscle activities and movement kinematics. With this model, we compared the characteristics of muscle activities for two movement tasks, a step-tracking task and a smooth pursuit task. In control subjects, the CNS adjusted two components of motor command (i.e., muscle activities) to meet the requirements of the tasks. For example, for the step-tracking task to stationary targets without any reference velocity, patterns of the muscle activities were correlated primarily with the position, with very low correlation with velocity (low *B_r_/K_r_* ratio). In contrast, for the smooth pursuit task in which the target moves with known velocity and position, the muscle activities were correlated comparably with the velocity and position of the target motion (higher *B_r_/K_r_* ratio). In contrast, the ability of cerebellar patients to select a proper *B_r_/K_r_* ratio depending on the task requirement was markedly deteriorated (Lee et al., 2015). Overall, *B_r_/K_r_* ratio provides a novel parameter to characterize the motor function of cerebellar patients.

When we analyzed the smooth pursuit movement in the previous study, we treated the movement of all frequencies as a whole, based on an assumption that there is a single controller operating at all frequencies. In the present study, however, we *reanalyzed* the same data to find that it actually contained *two* distinct components in different frequency ranges, i.e., F1 and F2 (Figure 3C,D). Therefore, we further employed the same method to evaluate each component separately, with the *B_r_/K_r_* ratio. The major F1 component belonged to the same lower frequency range as the target motion (Figure 3C, <0.5 Hz, F1 component) and encoded both velocity and position (i.e., higher *B_r_/K_r_* ratio) of the smooth target motion (Figure 4A). This composition of the F1 component appeared suitable to synchronize the wrist movement with the target motion in a *predictive* manner (Figure 6). In contrast, the minor F2 component belonged to a higher frequency range (Figure 3C, 0.5 Hz<, F2 component) and mostly encoded position (i.e., low *B_r_/K_r_* ratio) of small step-wise movements (Figure 4B). So far, we do not fully understand the functional roles of the F2 component. Nevertheless, the low *B_r_/K_r_* ratio of the F2 component suggests that the F2 component may provide small and intermittent positional corrections (i.e., feedback) during the pursuit movement. Indeed, the F2 component appeared to provide quick corrective (i.e., feedback) mechanism (Beppu et al., 1987; Miall et al., 1993a) and is recruited more when the precision of the F1 component is deteriorated (Figure 8D). In other words, the F1 and the F2 components appear to function cooperatively. We will focus on the nature of the F2 component and its cooperation with the F1 component in a separate paper. Overall, the *B_r_/K_r_* ratio again provides a unique tool to characterize functional significance of motor commands for goal directed movements.

### The *B_r_/K_r_* Ratios for F1 and F2 Components in Patients With Cerebellar Ataxia and the Role of the Cerebellum in Predictive Control

In contrast to the distinct *B_r_/K_r_* ratios for F1 and F2 components in control subjects mentioned above, the component-specific differences in the *B_r_/K_r_* ratio were much smaller in the cerebellar patients (Figure 5B). Indeed, the patients relied on position-dominant control even for the predictive F1 component (Figure 5B, 7B). In other words, they were not able to recruit the velocity-dominant control. These findings suggest that the cerebellum makes an important contribution to the predictive control of the pursuit movement, which is impaired in cerebellar ataxia. Our observations also explain why movements in cerebellar ataxia are characterized by a lack of smoothness. In contrast to control subjects, who achieve smooth movement with continuous velocity control (Figure 3A, *top inset*), cerebellar patients must rely on position-dominant step-wise movements (Figure 7A, *top inset*), which are probably manageable only with position control. The step-wise position-dominant movement appears to be a *default* mode of motor control that utilized by patients with cerebellar ataxia as a compensation method. Indeed, the low *B_r_/K_r_* ratio for the F2 component in cerebellar patients was similar to that in control subjects (Figure 5B, *F2*). On the other hand, velocity control is continuous and predictive in nature. Therefore, the impaired velocity control and decrease in tracking accuracy (Figure 8C) in these patients may suggest a deficit in prediction in cerebellar ataxia. It should be noted that the poor precision is not the only problem with the predictive control of the cerebellar patients. The prediction is delayed significantly more (∼100 ms) than in controls (Figure 6B). The delay itself may be simply explained as poor recruitment of output from the cerebellar nuclei due to decrease in *disinhibition* of output neurons (Ishikawa et al., 2014, 2015, 2016). The prediction that is delayed by this amount is no longer a prediction and may force the patients to depend on the *pure* feedback control *further destabilizing* the wrist movement ataxic as typically seen in Figure 7A.

### The Predictive (F1) Component of the Pursuit Movement and Precision of Motor Control

The *B_r_/K_r_* ratio reflects the composition of the motor command from the controller in the CNS. Considering the redundancy between muscle activities and movement kinematics, it is possible that different patterns of muscle activities could generate exactly the same movement kinematics. In other words, it is possible, at least theoretically, that accurate pursuit movement observed in the control subjects (Figure 3A, *top inset*) could be generated with muscle activities with even lower *B_r_/K_r_* ratios compared to those observed in cerebellar patients. Nevertheless, the *B_r_/K_r_* ratio of the F1 (*predictive*) component demonstrated a strong negative correlation with the error of the predictive movement (Figure 8A) and a strong positive correlation with the accuracy of the overall pursuit movement (Figure 8B). Therefore, the *B_r_/K_r_* ratio of the predictive (F1) component provides a unique parameter that represents accuracy of the predictive control for the pursuit movement in patients with ataxia.

### Quantitative Evaluations of the Motor Functions of Patients With Cerebellar Ataxia Based on the *B_r_/K_r_* Ratio

Precise evaluations of motor functions of patients with neurological disorders are essential for both monitoring the progress of disease and evaluation of effects of treatment. Although several groups have tried to perform quantitative evaluations of cerebellar ataxia with arm movements (Nakanishi et al., 1992; Sanguineti et al., 2003; Menegoni et al., 2009), their evaluations are mostly limited to movement kinematics. The authors have reported some features of movement kinematics, such as more curved and irregular hand paths, with a more asymmetric speed profile, in ataxic patients. However, movement kinematics cannot tell much about causal muscle activities or motor commands due to the redundancy of the musculoskeletal system. In other words, muscle activities provide more information than movement kinematics. Therefore, it is desirable to find anomalies of the motor commands directly (Diener and Dichgans, 1992; Manto, 1996) rather than the resultant movement anomalies. In this study, we evaluated the motor functions of patients with cerebellar ataxia based on the level of muscle activities (i.e., EMG signals). In particular, the decreased *B_r_/K_r_* ratio for the F1 component strongly reflected the pathophysiological changes in these patients (Figure 5B, 6B, 8). We will test this hypothesis by monitoring the *B_r_/K_r_* ratios for the F1 component of the pursuit task in ataxic patients for a long period.

## Conclusion

In conclusion, the *B_r_/K_r_* ratio of the F1 component provides a unique parameter to characterize the accuracy in terms of predictive control of voluntary goal-directed motion. This method can be applied in the numerous forms of cerebellar ataxias encountered in daily practice.

## Ethics Statement

This study was carried out in accordance with the recommendations of the ethics committees of the Tokyo Metropolitan Institute of Medical Science, Tokyo Metropolitan Neurological Hospital, and Tokyo Medical University with written informed consent from all subjects. All subjects gave written informed consent in accordance with the Declaration of Helsinki. The protocol was approved by the ethics committees of the Tokyo Metropolitan Institute of Medical Science, Tokyo Metropolitan Neurological Hospital, and Tokyo Medical University.

## Author Contributions

SK and JL conceived and designed the experiments. JL, SK, and HM conducted the experiments. JL, SK, HM, and HT analyzed the data. SK, JL, HM, HT, MM, and CH wrote the manuscript.

## Conflict of Interest Statement

The authors declare that the research was conducted in the absence of any commercial or financial relationships that could be construed as a potential conflict of interest.

## References

[B1] Bastian A. J. (2006). Learning to predict the future: the cerebellum adapts feedforward movement control. *Curr. Opin. Neurobiol.* 16 645–649. 10.1016/j.conb.2006.08.016 17071073

[B2] Beppu H. Nagaoka M. Tanaka R. (1987). Analysis of cerebellar motor disorders by visually-guided elbow tracking movement. 2. Contribution of the visual cues on slow ramp pursuit. *Brain* 110 1–18. 10.1093/brain/110.1.1 3801845

[B3] Blakemore S. J. Frith C. D. Wolpert D. M. (2001). The cerebellum is involved in predicting the sensory consequences of action. *Neuroreport* 12 1879–1884. 10.1097/00001756-200107030-00023 11435916

[B4] Crevecoeur F. Kurtzer I. (2018). Long-latency reflexes for inter-effector coordination reflect a continuous state feedback controller. *J. Neurophysiol.* 120 2466–2483. 10.1152/jn.00205.2018 30133376

[B5] Crevecoeur F. Munoz D. P. Scott S. H. (2016). Dynamic multisensory integration: somatosensory speed trumps visual accuracy during feedback control. *J. Neurosci.* 36 8598–8611. 10.1523/JNEUROSCI.0184-16.2016 27535908 PMC6601898

[B6] Davidson P. R. Wolpert D. M. (2005). Widespread access to predictive models in the motor system: a short review. *J. Neur. Eng.* 2:S313. 16135891 10.1088/1741-2560/2/3/S11

[B7] Desmurget M. Grafton S. (2000). Forward modeling allows feedback control for fast reaching movements. *Trends Cogn. Sci.* 4 423–431. 10.1016/s1364-6613(00)01537-0 11058820

[B8] Diener H. C. Dichgans J. (1992). Pathophysiology of cerebellar ataxia. *Mov. Disord.* 7 95–109. 10.1002/mds.870070202 1584245

[B9] Ebner T. J. Pasalar S. (2008). Cerebellum predicts the future motor state. *Cerebellum* 7 583–588. 10.1007/s12311-008-0059-3 18850258 PMC2754147

[B10] Franklin D. W. Franklin S. Wolpert D. M. (2014). Fractionation of the visuomotor feedback response to directions of movement and perturbation. *J. Neurophysiol.* 112 2218–2233. 10.1152/jn.00377.2013 25098965 PMC4274920

[B11] Franklin S. Wolpert D. M. Franklin D. W. (2017). Rapid visuomotor feedback gains are tuned to the task dynamics. *J. Neurophysiol.* 118 2711–2726. 10.1152/jn.00748.2016 28835530 PMC5672538

[B12] Härdle W. K. Simar L. (2003). “Canonical correlation analysis,” in *Applied Multivariate Statistical Analysis* (New York, NY: Springer), 321–330.

[B13] Hoffman D. S. Strick P. L. (1999). Step-tracking movements of the wrist. IV. Muscle activity associated with movements in different directions. *J. Neurophysiol.* 81 319–333. 10.1152/jn.1999.81.1.319 9914292

[B14] Ishikawa T. Kakei S. Mitoma H. (2015). Overlooked Holmes’ clinical signs: reevaluation by recent physiological findings. *Cerebellum Ataxias* 2:13. 10.1186/s40673-015-0033-z 26550482 PMC4636821

[B15] Ishikawa T. Tomatsu S. Izawa J. Kakei S. (2016). The cerebro-cerebellum: could it be loci of forward models? *Neurosci. Res.* 104 72–79. 10.1016/j.neures.2015.12.003 26704591

[B16] Ishikawa T. Tomatsu S. Tsunoda Y. Lee J. Hoffman D. S. Kakei S. (2014). Releasing dentate nucleus cells from purkinje cell inhibition generates output from the cerebrocerebellum. *PLoS One* 9:e108774. 10.1371/journal.pone.0108774 25279763 PMC4184834

[B17] Kakei S. Hoffman D. S. Strick P. L. (1999). Muscle and movement representations in the primary motor cortex. *Science* 285 2136–2139. 10.1126/science.285.5436.2136 10497133

[B18] Kakei S. Hoffman D. S. Strick P. L. (2003). Sensorimotor transformations in cortical motor areas. *Neurosci. Res.* 46 1–10. 10.1016/s0168-0102(03)00031-2 12725907

[B19] Kawato M. (1999). Internal models for motor control and trajectory planning. *Curr. Opin. Neurobiol.* 9 718–727. 10.1016/s0959-4388(99)00028-8 10607637

[B20] Kawato M. Kuroda T. Imamizu H. Nakano E. Miyauchi S. Yoshioka T. (2003). Internal forward models in the cerebellum: fMRI study on grip force and load force coupling. *Prog. Brain Res.* 142 171–188. 10.1016/s0079-6123(03)42013-x 12693261

[B21] Koike Y. Kawato M. (1995). Estimation of dynamic joint torques and trajectory formation from surface electromyography signals using a neural network model. *Biol. Cybern.* 73 291–300. 10.1007/s004220050185 7578470

[B22] Lacquaniti F. Licata F. Soechting J. F. (1982). The mechanical behavior of the human forearm in response to transient perturbations. *Biol. Cybern.* 1982 35–46. 10.1007/bf00353954 7093368

[B23] Lee J. Kagamihara Y. Kakei S. (2008). Quantitative evaluation of movement disorders in neurological diseases based on EMG signals. *Conf. Proc. IEEE Eng. Med. Biol. Soc.* 2008 181–184. 10.1109/IEMBS.2008.4649120 19162623

[B24] Lee J. Kagamihara Y. Kakei S. (2013). Quantitative evaluation of cerebellar ataxia based on pathological patterns of the muscle activities. *Conf. Proc. IEEE Eng. Med. Biol. Soc.* 2013 902–905. 10.1109/EMBC.2013.6609647 24109834

[B25] Lee J. Kagamihara Y. Kakei S. (2015). A new method for functional evaluation of motor commands in patients with cerebellar ataxia. *PLoS One* 10:e0132983. 10.1371/journal.pone.0132983 26186225 PMC4505901

[B26] Lee J. Kagamihara Y. Tomatsu S. Kakei S. (2012). The functional role of the cerebellum in visually guided tracking movement. *Cerebellum* 11 426–433. 10.1007/s12311-012-0370-x 22396331

[B27] Lesage E. Morgan B. E. Olson A. C. Meyer A. S. Miall R. C. (2012). Cerebellar rTMS disrupts predictive language processing. *Curr. Biol.* 2012 R794–R795. 23017990 10.1016/j.cub.2012.07.006 PMC3459089

[B28] Mannard A. Stein R. B. (1973). Determination of the frequency response of isometric soleus muscle in the cat using random nerve stimulation. *J. Physiol.* 229 275–296. 10.1113/jphysiol.1973.sp010138 4353409 PMC1350307

[B29] Manto M. (1996). Pathophysiology of cerebellar dysmetria: the imbalance between the agonist and the antagonist electromyographic activities. *Eur. Neurol.* 36 333–337. 10.1159/000117289 8954299

[B30] Menegoni F. Milano E. Trotti C. Galli M. Bigoni M. Baudo S. (2009). Quantitative evaluation of functional limitation of upper limb movements in subjects affected by ataxia. *Eur. J. Neurol.* 16 232–239. 10.1111/j.1468-1331.2008.02396.x 19146643

[B31] Miall R. C. Christensen L. O. Cain O. Stanley J. (2007). Disruption of state estimation in the human lateral cerebellum. *PLoS Biol.* 5:e316. 10.1371/journal.pbio.0050316 18044990 PMC2229864

[B32] Miall R. C. Weir D. J. Stein J. F. (1993a). Intermittency in human manual tracking tasks. *J. Mot. Behav.* 25 53–63. 10.1080/00222895.1993.9941639 12730041

[B33] Miall R. C. Weir D. J. Wolpert D. M. Stein J. F. (1993b). Is the cerebellum a smith predictor? *J. Mot. Behav.* 25 203–216. 10.1080/00222895.1993.9942050 12581990

[B34] Miall R. C. Wolpert D. M. (1996). Forward models for physiological motor control. *Neural Netw.* 9 1265–1279. 10.1016/s0893-6080(96)00035-4 12662535

[B35] Mitoma H. Adhikari K. Aeschlimann D. Chattopadhyay P. Hadjivassiliou M. Hampe C. S. (2016). Consensus paper: neuroimmune mechanisms of cerebellar ataxias. *Cerebellum* 15 213–232. 10.1007/s12311-015-0664-x 25823827 PMC4591117

[B36] Nakanishi R. Yamanaga H. Okumura C. Murayama N. Ideta T. (1992). A quantitative analysis of ataxia in the upper limbs. *Rinsho Shinkeigaku* 32 251–258. 1628447

[B37] Nowak D. A. Topka H. Timmann D. Boecker H. Hermsdörfer J. (2007). The role of the cerebellum for predictive control of grasping. *Cerebellum* 6 7–17. 10.1080/14734220600776379 17366262

[B38] Pasalar S. Roitman A. V. Durfee W. K. Ebner T. J. (2006). Force field effects on cerebellar purkinje cell discharge with implications for internal models. *Nat. Neurosci.* 9 1404–1411. 10.1038/nn1783 17028585

[B39] Popa L. S. Ebner T. J. (2019). Cerebellum, predictions and errors. *Front. Cell Neurosci.* 12:524 10.3389/fncel.2018.00524 PMC6340992 30697149

[B40] Pruszynski J. A. Kurtzer I. Nashed J. Y. Omrani M. Brouwer B. Scott S. H. (2011). Primary motor cortex underlies multi-joint integration for fast feedback control. *Nature* 478 387–390. 10.1038/nature10436 21964335 PMC4974074

[B41] Pruszynski J. A. Omrani M. Scott S. H. (2014). Goal-dependent modulation of fast feedback responses in primary motor cortex. *J. Neurosci.* 34 4608–4617. 10.1523/JNEUROSCI.4520-13.2014 24672006 PMC6608123

[B42] Sanguineti V. Morasso P. G. Baratto L. Brichetto G. Luigi Mancardi G. Solaro C. (2003). Cerebellar ataxia: quantitative assessment and cybernetic interpretation. *Hum. Mov. Sci.* 22 189–205. 10.1016/s0167-9457(02)00159-8 12667749

[B43] Schlerf J. Ivry R. B. Diedrichsen J. (2012). Encoding of sensory prediction errors in the human cerebellum. *J. Neurosci.* 32 4913–4922. 10.1523/JNEUROSCI.4504-11.2012 22492047 PMC4332713

[B44] Scott S. H. Cluff T. Lowrey C. R. Takei T. (2015). Feedback control during voluntary motor actions. *Curr. Opin. Neurobiol.* 33 85–94. 10.1016/j.conb.2015.03.006 25827274

[B45] Shadmehr R. Krakauer J. W. (2008). A computational neuroanatomy for motor control. *Exp. Brain Res.* 185 359–381. 10.1007/s00221-008-1280-5 18251019 PMC2553854

[B46] Shadmehr R. Smith M. A. Krakauer J. W. (2010). Error correction, sensory prediction, and adaptation in motor control. *Ann. Rev. Neurosci.* 33 89–108. 10.1146/annurev-neuro-060909-153135 20367317

[B47] Shin D. Kim J. Koike Y. (2009). A myokinetic arm model for estimating joint torque and stiffness from EMG signals during maintained posture. *J. Neurophysiol.* 101 387–401. 10.1152/jn.00584.2007 19005007

[B48] Soechting J. F. Lacquaniti F. (1988). Quantitative evaluation of the electromyographic responses to multidirectional load perturbations of the human arm. *J. Neurophysiol.* 59 1296–1313. 10.1152/jn.1988.59.4.1296 3373279

[B49] Standenmann D. Roeleveld K. Stegeman D. F. van Dieën J. H. (2010). Methodological aspects of SEMG recordings for force estimation – a tutorial and review. *J. Electromyogr. Kinesiol.* 20 375–387. 10.1016/j.jelekin.2009.08.005 19758823

[B50] Synofzik M. Lindner A. Thier P. (2008). The cerebellum updates predictions about the visual consequences of one’s behavior. *Curr. Biol.* 18 814–818. 10.1016/j.cub.2008.04.071 18514520

[B51] Tanaka H. Ishikawa T. Kakei S. (2019). Neural evidence of the cerebellum as a state predictor. *Cerebellum* 10.1007/s12311-018-0996-4 [Epub ahead of print]. 30627965 PMC6517560

[B52] Todorov E. Jordan M. I. (2002). Optimal feedback control as a theory of motor coordination. *Nat. Neurosci.* 5 1226–1235. 10.1038/nn963 12404008

[B53] Tseng Y. W. Diedrichsen J. Krakauer J. W. Shadmehr R. Bastian A. J. (2007). Sensory prediction errors drive cerebellum-dependent adaptation of reaching. *J. Neurophysiol.* 98 54–62. 10.1152/jn.00266.2007 17507504

[B54] Wolpert D. M. Ghahramani Z. Jordan M. I. (1995). An internal model for sensorimotor integration. *Science* 269 1880–1882. 10.1126/science.7569931 7569931

[B55] Wolpert D. M. Miall R. C. Kawato M. (1998). Internal models in the cerebellum. *Trends Cogn. Sci.* 2 338–347. 10.1016/s1364-6613(98)01221-2 21227230

